# The cortical modulation of stimulus-specific adaptation in the auditory midbrain and thalamus: a potential neuronal correlate for predictive coding

**DOI:** 10.3389/fnsys.2015.00019

**Published:** 2015-03-09

**Authors:** Manuel S. Malmierca, Lucy A. Anderson, Flora M. Antunes

**Affiliations:** ^1^Auditory Neuroscience Laboratory, Institute of Neuroscience of Castilla y León (INCyL), University of SalamancaSalamanca, Spain; ^2^Faculty of Medicine, Department of Cell Biology and Pathology, University of SalamancaSalamanca, Spain

**Keywords:** auditory, IC, MGB, SSA, MMN, corticofugal projections, cooling technique, predictive coding

## Abstract

To follow an ever-changing auditory scene, the auditory brain is continuously creating a representation of the past to form expectations about the future. Unexpected events will produce an error in the predictions that should “trigger” the network’s response. Indeed, neurons in the auditory midbrain, thalamus and cortex, respond to rarely occurring sounds while adapting to frequently repeated ones, i.e., they exhibit stimulus specific adaptation (SSA). SSA cannot be explained solely by intrinsic membrane properties, but likely involves the participation of the network. Thus, SSA is envisaged as a high order form of adaptation that requires the influence of cortical areas. However, present research supports the hypothesis that SSA, at least in its simplest form (i.e., to frequency deviants), can be transmitted in a bottom-up manner through the auditory pathway. Here, we briefly review the underlying neuroanatomy of the corticofugal projections before discussing state of the art studies which demonstrate that SSA present in the medial geniculate body (MGB) and inferior colliculus (IC) is not inherited from the cortex but can be modulated by the cortex via the corticofugal pathways. By modulating the gain of neurons in the thalamus and midbrain, the auditory cortex (AC) would refine SSA subcortically, preventing irrelevant information from reaching the cortex.

## Introduction

Sounds seldom occur in isolation and we are constantly swamped with a cacophony of sounds that impinge on our ears at every instant, therefore, an essential operation of the brain is to detect rare and potentially important stimuli while ignoring irrelevant ambient backgrounds (Ranganath and Rainer, 2003; Kaya and Elhilali, 2014). Since we are living in a dynamic and permanently changing world, to organize the auditory scene the brain needs to “*adapt*” and efficiently respond to changes in the stimulus incidence and context. Adaptation is an omnipresent property of neurons in the auditory system, however, most types of adaptation previously described in the literature are governed by activity-dependent mechanisms operating at the level of the neuron’s output rather than its input, such as those dependent on the history of the stimulation (Calford and Semple, 1995; Brosch and Schreiner, 1997; Ingham and McAlpine, 2004; Furukawa et al., 2005; Gutfreund and Knudsen, 2006; Gutfreund, 2012). The so-called stimulus-specific adaptation (SSA) is a higher level of adaptation which results from adaptation to a specific stimulus, rather than from the intrinsic properties of the neuron (Ulanovsky et al., 2003, 2004). Neurons showing SSA adapt to frequently occurring stimuli (standards) yet respond strongly to rare stimuli (deviants) (Dragoi et al., 2000; Ulanovsky et al., 2003; Katz et al., 2006; Reches and Gutfreund, 2008; Anderson et al., 2009; Malmierca et al., 2009, 2014; von der Behrens et al., 2009; Antunes et al., 2010; Pérez-González and Malmierca, 2012, 2014; Escera and Malmierca, 2014; Nelken, 2014). Such deviant stimuli, i.e., those that are novel in time and space, are perceptually advantaged and give rise to psychophysical effects such as attention capture (Tiitinen et al., 1994) or pop-outs (Diliberto et al., 2000). However, in order to ascertain that a specific stimulus is novel, there must be a neuronal network capable of comparing current and previous stimuli, as shown by the computational studies of Abbott et al. (1997) and Eytan et al. (2003). Thus, at the neuronal level, neurons showing SSA must integrate sensory information to create a predictive model of the world, enabling them to adapt to commonly occurring stimuli and respond more strongly to novel features in the environment. In other words, the neuron’s previous experience determines its future sensitivity, which suggests SSA may be a basic mechanism underlying predictive coding (Friston, 2005; Baldeweg, 2006; Bar, 2007; Winkler et al., 2009; Bendixen et al., 2012). Moreover, previous studies have also suggested that SSA could be linked to auditory memory, recognition of acoustic objects and auditory scene analysis (Nelken, 2004; Winkler et al., 2009).

**Figure 1:**
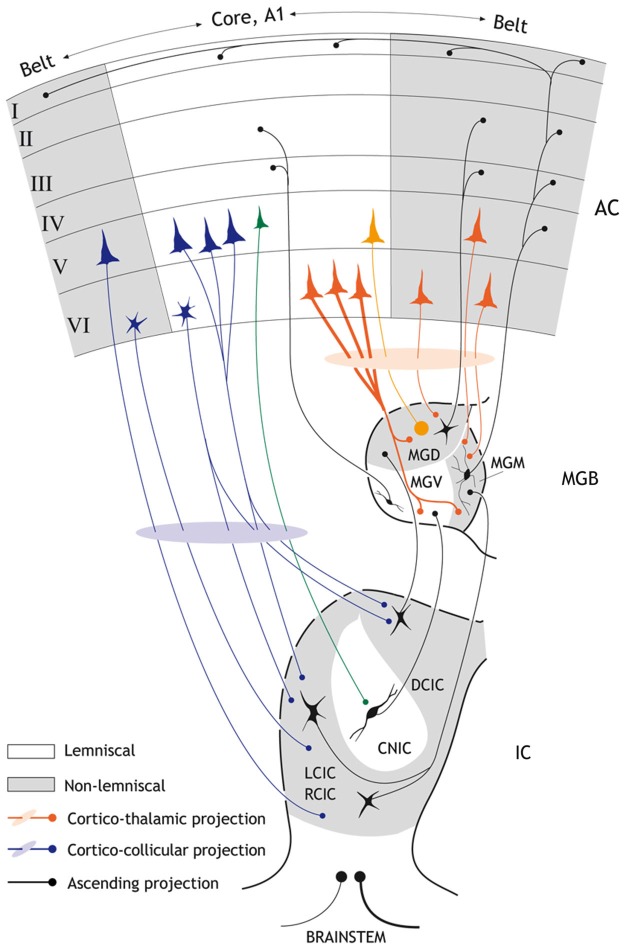
**Schematic diagram showing the major anatomical subdivisions of the IC, MGB and AC that illustrates the ascending and descending pathways from the midbrain up to the cortex and back**. Non-lemniscal (belt) divisions are highlighted as gray areas. Black connections indicate ascending projections; while red connections indicate major cortico-thalamic connections and purple connections major cortico-collicular projections. Strong SSA is mainly restricted to “*non-lemniscal*” regions of the IC and MGB, but is found in the “*lemniscal*” or core A1. The major cortico-collicular projections emerge from pyramidal neurons in layer V (but a few small neurons deep in layer VI also contribute to this pathway and project predominantly to the collicular cortices). The larger pyramidal neurons from layer V project to the cortical regions of the IC while the smaller pyramidal neurons from layer V project to the CNIC (green). By contrast, the major cortico-thalamic projections emerge from pyramidal neurons in layer VI (but a few pyramidal neurons from layer V also contribute to this pathway). Most terminal boutons arising from the AC and terminating in the MGB are small (~0.5 μm^2^ in diameter) and most likely originate from the pyramidal neurons of layer VI. Some cortico-thalamic terminals boutons arise from layer V (orange) and are very large (>2 μm^2^). There are also interactions between the core and belt areas of the AC (horizontal black arrows). The connections between the reticular thalamic nucleus, MGB and AC as well as the contralateral corticofugal projections are not shown for simplicity. Abbreviations: A1, primary auditory cortex; AC auditory cortex; CNIC, central nucleus of the inferior colliculus; DCIC, dorsal cortex of the inferior colliculus; LCIC, RCIC; lateral and rostral cortex of the inferior colliculus; MGD; dorsal division of the medial geniculate body; MGM; medial division of the medial geniculate body; MGV; ventral division of the medial geniculate body.

**Figure 2:**
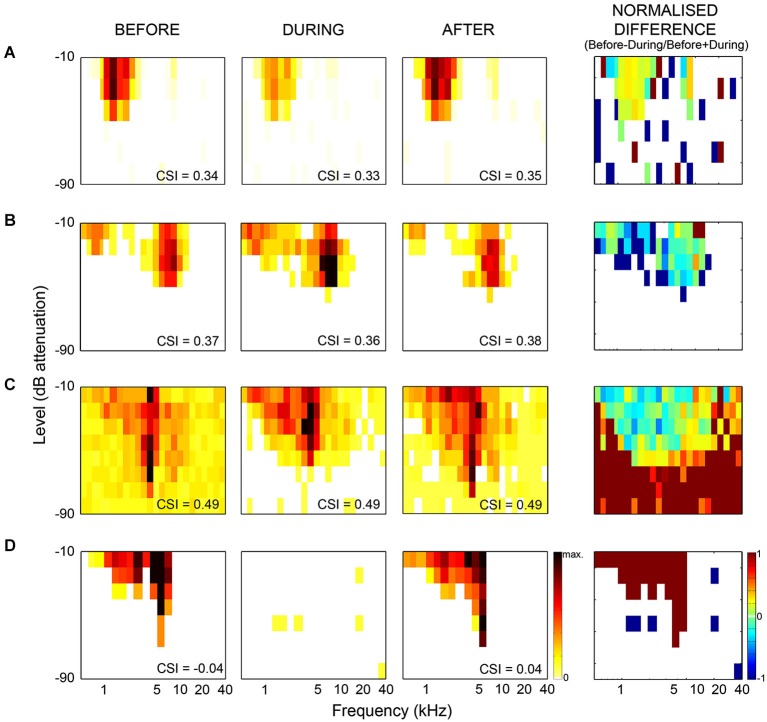
**Examples of frequency response areas in the inferior colliculus recorded from neurons which showed no change in SSA during cooling**. The CSI index quantifies the level of SSA, and was calculated as CSI = [d(f1) + d(f2) − s(f1) +s(f2)] / [d(f1) + d(f2) + s(f1) + s(f2)], where d(fi) and s(fi) were responses (in spike counts/stimulus) to either frequency fi when it was deviant or standard, respectively. The CSI index was maintained, but there were significant changes in firing rate, spontaneous activity and latencies of the neurons. **(A)** example showing decreased firing rate during cooling. **(B)** increased firing rate during cooling. **(C)** example showing decreased spontaneous rate, increased threshold and increased firing within the frequency response area (FRA) during cooling. **(D)** example which ceased firing during cooling. First column shows FRA before cooling, second column shows FRA during cooling, third column shows FRA after cooling. Firing rate indicated by color bar to right of “After” FRA in **(D)**. The min-max firing rate range is the same across each neuron for all conditions, although varies between neurons. The fourth column shows the normalized difference in FRA (before-during/before+during), difference in firing indicated by color bar to right of difference plot in **(D)** (cool colors = −1, hot colors = 1, no firing = white). Redrawn and modified from Anderson and Malmierca (2013).

In the auditory brain, SSA occurs in the midbrain (inferior colliculus, IC), thalamus (medial geniculate body, MGB) and cortex (Kraus et al., 1994; King et al., 1995; Ulanovsky et al., 2003, 2004; Pérez-González et al., 2005; Reches and Gutfreund, 2008; Anderson et al., 2009; Malmierca et al., 2009, 2014; von der Behrens et al., 2009; Yu et al., 2009; Antunes et al., 2010; Reches et al., 2010; Taaseh et al., 2011; Zhao et al., 2011; Patel et al., 2012; Pérez-González and Malmierca, 2012, 2014; Hershenhoren et al., 2014; Nelken, 2014). Evidence for SSA in the brainstem has not been extensively investigated; however neurons within the cochlear nucleus do not appear to exhibit SSA in response to similar paradigms that would elicit SSA in the midbrain (Ayala et al., 2013). SSA is strong in the non-lemniscal subcortical regions of the IC and MGB (Anderson et al., 2009; Malmierca et al., 2009; Antunes et al., 2010), but the primary auditory cortex (A1) is the first lemniscal station where SSA seems to be widespread and strong (Ulanovsky et al., 2003). Thus, SSA was originally suggested to emerge in the auditory cortex (AC) as a high order feature of sensory processing that would be transmitted to subcortical nuclei in a top-down fashion (Nelken and Ulanovsky, 2007). Indeed, it is well known that a remarkable feature of the thalamus is the massive set of corticofugal projections that it receives (Figure 1). In the MGB, these projections outnumber the ascending projections by a factor of 10 (Winer et al., 2001; Kimura et al., 2003, 2005, 2007; Winer, 2006; Winer and Lee, 2007; Ojima and Rouiller, 2011) and strongly modulate the responses of MGB neurons (Ryugo and Weinberger, 1976; Villa et al., 1991, 1999; He et al., 2002; He, 2003a,b; Palmer et al., 2007). Similarly, the IC in the midbrain also receives a significant corticofugal projection (Saldaña et al., 1996; Bajo et al., 2007; Stebbings et al., 2014) which although not as heavy and dense as the MGB, has been demonstrated to have a strong influence on the collicular neuronal responses (Yan and Suga, 1998; Jen et al., 2001; Yan and Ehret, 2001, 2002; Jen and Zhou, 2003; Yan et al., 2005; Nakamoto et al., 2008, 2010; Markovitz et al., 2013; Figures 1, 2).

**Figure 3:**
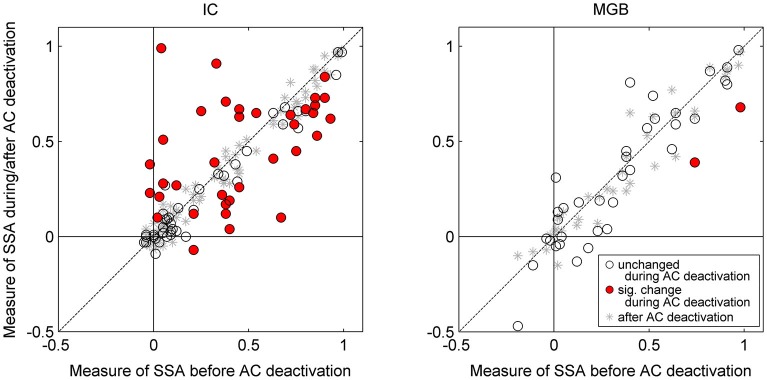
**Scatterplots for IC neurons (left panel) and MGB neurons (right panel) of CSI before cooling vs. CSI during cooling (circles) and after cooling (asterisks)**. Red circles indicate those neurons which show a significant change with cooling, whereas those with open circles indicate a non-significant change. All neurons included in the analyses returned to their previous CSI values after cooling (all asterisks lay along the line of equality for before vs. after cooling condition). Auditory cortical deactivation could have one of three effects on the SSA sensitivity of IC neurons; CSI values either showed no change, or a significant decrease or increase. By contrast, CSI values in the MGB were unchanged during cortical cooling (with the exception of two neurons which showed a significant decrease in SSA). Data plotted from Antunes and Malmierca (2011), and Anderson and Malmierca (2013).

**Figure 4:**
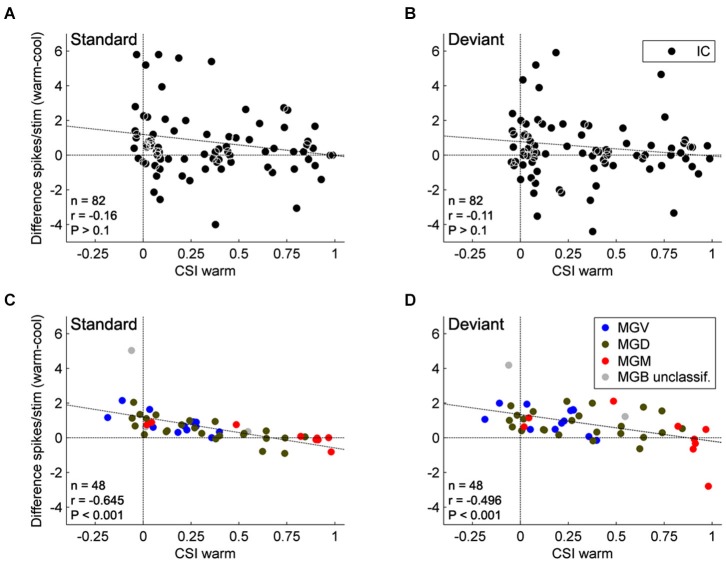
**Effect of AC deactivation on the firing rate of IC and MGB neurons**. Scatterplots of the CSI (warm condition) vs. the difference in firing rate between the warm and cool conditions (spikes/stimulus difference) in response to standard (**A**, IC; **C**, MGB), and deviant stimuli (**B**, IC; **D**, MGB), for each neuron. Black dots represent neurons in the IC (**A,B**; ***n* = 82**), while blue, green, and red dots represent the neurons that were localized to the ventral (*n* = 12), dorsal (*n* = 24), and medial (*n* = 9) subdivisions of the MGB, respectively (total *n* = 45, neurons that were localized to one of the three MGB subdivisions). Positive values indicate a reduction in firing rate with AC deactivation; and negative values an increment (above and below the horizontal line at the origin, respectively). Note that no correlation is shown for the IC neurons, while MGB neurons show a significant negative correlation. These data suggest that the gain exerted by the AC on MGB neurons depends on the level of SSA that the MGB neurons show. There was no effect of subdivision nor was there an interaction between condition and subdivision (*n* = 45, Two-way repeated measures ANOVA, for the responses to the deviants: *F*_(1,42)_ = 21.95, *P* < 0.001, main effect of condition; *F*_(2,42)_ = 2.96, *P* = 0.06, main effect of subdivision; and *F*_(2,42)_ = 0.12, *P* = 0.89, interaction; Two way repeated measures ANOVA, for the responses to the standards: *F*_(1,42)_ = 22.88, *P* < 0.001, main effect of condition; *F*_(2,42)_ = 2.89, *P* = 0.07, main effect of subdivision; and *F*_(2,42)_ = 1.06, *P* = 0.36, interaction). Data plotted from Antunes and Malmierca (2011), and Anderson and Malmierca (2013).

Here, we will focus on the effect of the descending cortical projections on SSA at the level of the MGB and IC (Figures 2–4). By disentangling the effect of cortical influence on subcortical SSA we endeavor to gain a better understanding of the neuronal circuitry underlying this property. We begin with a brief introduction to the descending *cortico-collicular* and *cortico-thalamic* projections before detailing our recent studies using cortical-cooling to study the effect of reversibly deactivating the AC on SSA in the IC and MGB.

## The descending pathway from the auditory cortex to the thalamus and midbrain

In parallel to the ascending auditory pathways, there are stepwise, descending projections from the AC to the organ of Corti (Malmierca and Ryugo, 2011; Malmierca, 2015). Although these corticofugal pathways have been known since the end of the 19th century (Held, 1893), a renaissance in their study was triggered by the description of the olivocochlear bundle in 1946 by Rasmussen (1946, 1953). The AC projects to a wide range of subcortical targets in the auditory pathway (Winer, 2006; Winer and Lee, 2007), the largest of which are to the MGB (auditory thalamus; Figure 1) and the IC (midbrain; Figure 1).

The *cortico-thalamic system* (Figure 1) is the heaviest projection of the corticofugal network, not only in the descending auditory system, but of the whole brain, comparable only to the corticospinal tract (Winer et al., 2001; Winer, 2006; Malmierca and Ryugo, 2011). The cortico-thalamic system forms a reciprocal connection between the cortex and the thalamus, with a large-scale topographical overlap in the spatial territories of thalamocortical cells and corticothalamic axonal terminals (Winer, 2006, but see Llano and Sherman, 2008). Most terminal boutons arising from the AC and terminating in the MGB are small (~0.5 μm^2^ in diameter) and most likely originate from the pyramidal neurons of layer VI (Bartlett et al., 2000), but a few very large boutons (>2 μm^2^) also occur and are thought to originate from neurons in layer V (Rouiller and Welker, 1991, 2000; Shi and Cassell, 1997; Bartlett et al., 2000). These large corticothalamic terminals tend to form complexes with the dendrites partially surrounded by astrocytic processes (Bartlett et al., 2000). Sherman and Guillery (1998) proposed the notion of “drivers” and “modulators” of thalamic neurons in the visual and somatosensory thalamus; this hypothesis has since been applied to the auditory system (Llano and Sherman, 2008). According to this theory, type I terminals play a modulatory role in the first-order thalamic nuclei, such as the ventral subdivision of the MGB (MGV). Thus, the corticothalamic inputs converge with ascending inputs on thalamic neurons such that the ascending inputs drive the thalamic neurons, and the cortical inputs modulate them. In contrast, in “higher order” thalamic nuclei, such as the dorsal subdivision of the MGB (MGD), the “driver” inputs arise from the large type II axons and terminals originating from the AC, and interact with ascending input from the IC.

The major corticofugal projections are glutamatergic (Potashner et al., 1988) suggesting an excitatory function. The AC also projects to the auditory sector of the reticular thalamic nucleus, which in turns projects to the MGB (Rouiller and Welker, 1991, 2000; Bartlett et al., 2000) thus, providing the MGB with an inhibitory influence (Bartlett et al., 2000). Therefore, the corticofugal projection may play a key role in modulating the MGB responses to sound through a direct excitatory pathway and/or an indirect inhibitory pathway. Physiological studies based on electrical stimulation or cooling of the AC have confirmed the excitatory and inhibitory effects of the AC on MGB neurons (Ryugo and Weinberger, 1976; He, 1997, 2003a,b; He et al., 2002; Yu et al., 2004).

The *cortico-collicular system* (Figure 1) on the other hand, is made of projections that originate in the AC, bypass the MGB and terminate in the IC (Faye-Lund, 1985; Herbert et al., 1991; Saldaña et al., 1996; Winer et al., 1998; Doucet et al., 2003; Bajo and Moore, 2005; Bajo et al., 2007). Most of these studies have shown a topographic (tonotopic) organization of these projections arising from the primary AC (A1), such that the low frequency regions of A1 project to the dorsolateral region of the IC and the high frequency region of A1 projects to the ventromedial region of the IC. The projections originate bilaterally in multiple cortical areas (reviewed by Winer, 2005); however, the projections originating from A1 constitute the heaviest projection. Projections to the IC also originate from non-primary areas. These latter projections are more variable and less dense than those arising from A1 (Herbert et al., 1991; Bajo et al., 2007). The projections from A1 target the collicular cortices (dorsal, lateral and rostral cortex of the IC; DCIC, LCIC, and RCIC respectively) bilaterally, with the ipsilateral projection being most dense. The central nucleus of the IC also receives a weak, yet significant projection (Saldaña et al., 1996; Winer et al., 1998; Bajo et al., 2007; Nakamoto et al., 2013a,b); the projection to the central nucleus of the IC differs not only in the density of terminal boutons (which is always lower for the central nucleus), but also in their morphology, having, on average, thinner axons and smaller boutons than those terminating in the collicular cortices (Saldaña et al., 1996; Bajo et al., 2007).

The cortico-collicular projections originate primarily in layer V, and to a lesser extent in layer VI (Wong and Kelly, 1981; Games and Winer, 1988; Winer and Prieto, 2001; Doucet et al., 2003; Bajo and Moore, 2005; Bajo et al., 2007; Bajo and King, 2013; Stebbings et al., 2014). More detailed studies have shown that neurons from layer V include pyramidal cells, but those from layer VI are of unknown type except that they are described as “small labeled neurons deep in layer VI” (Bajo and Moore, 2005; Bajo and King, 2013). The largest population of these pyramidal neurons projects to the ipsilateral IC, and a smaller population projects to the contralateral IC or bilaterally to both ICs. Moreover, it seems that the larger pyramidal neurons from layer V project to the cortical regions of the IC while the smaller pyramidal neurons project to the central nucleus. Layer VI neurons seem to project primarily to the collicular cortices (Schofield, 2009). Furthermore, the pyramidal neurons involved in the descending pathway to the IC may correspond to intrinsic bursting neurons (Hefti and Smith, 2000; Slater et al., 2013).

The corticocofugal projection to the IC is glutamatergic (Feliciano and Potashner, 1995), thus the AC may modulate the processing of sounds in the IC either directly, or through the activation of local inhibitory connections within the IC. In this respect, it is worth mentioning that Mitani et al. (1983) found IPSPs in IC neurons after AC stimulation, and Nakamoto et al. (2013a,b) found a very small percentage of corticocollicular targets that were GABAergic IC neurons. These studies suggest that polysynaptic network mechanisms are necessary to achieve local inhibition in the IC after AC stimulation.

## The effect of the corticofugal projections on thalamic SSA

The AC has been shown to modulate several features of auditory processing in all subcortical regions including the MGB. For example, studies based on electrical stimulation of the AC have shown that the AC can facilitate or suppress responses in the MGB (He, 2003a,b). Furthermore, earlier studies using reversible AC deactivation (Ryugo and Weinberger, 1976; Villa et al., 1991) confirmed that the responses of many MGB neurons are under the control of the AC, thus it would not be surprising that the AC might influence SSA. A simple and elegant way to test this issue is to reversibly deactivate the cortex using the cooling technique (e.g., Lomber, 1999; Lomber et al., 1999, 2007; Lomber and Malhotra, 2008; Nakamoto et al., 2008, 2010; Carrasco and Lomber, 2009a,b, 2010; Coomber et al., 2011). Using this technique, we reversibly deactivated the AC to silence its neurons and the ipsilateral descending projections to the MGB (Antunes and Malmierca, 2011, 2014) in order to analyze what effect the AC exerts on the SSA exhibited by auditory thalamic neurons. Using an oddball stimulus designed to elicit SSA to frequencies (Ulanovsky et al., 2003, 2004; Malmierca et al., 2009; Antunes et al., 2010), we recorded single neuron responses throughout the MGB before, during and after reversibly deactivating the AC by cooling (Antunes and Malmierca, 2011, 2014).

Our results demonstrate that some general properties of the MGB responses were significantly modified during the period of cortical deactivation (such as frequency response maps, spontaneous activity, latency, etc. Figure 2 shows similar effects observed in the IC). This confirms the MGB receives strong cortical modulation (like other thalamic and subcortical nuclei) through the corticofugal pathway, as demonstrated in previous studies of the auditory (Ryugo and Weinberger, 1976; Villa et al., 1991, 1999; Bajo et al., 1995; Yu et al., 2004; Luo et al., 2008; Liu et al., 2010), visual (Sillito et al., 1994; Rushmore et al., 2005) and somatosensory systems (Ghosh et al., 1994). However, remarkably, despite changes in basic neuronal activity in the MGB during the period of AC deactivation, SSA levels and its dynamics over time were mostly unaffected (Figures 3, 4; Antunes and Malmierca, 2011, 2014).

**Figure 5:**
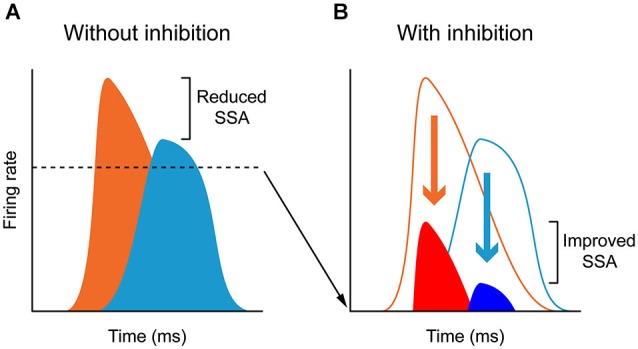
**Inhibition and “iceberg effect”:** In the absence of inhibition **(A)**, neurons respond to deviants (orange) and standards (light blue) with high firing rates and thus the deviant to standard ratio is small. By contrast, GABA_A_- mediated inhibition reduces the responses to both deviants (red) and standards (dark blue) acting as in the “iceberg effect”, thus increasing the deviant to standard ratio and enhancing SSA **(B)**. For more details, see Pérez-González et al. (2012).

The AC modulates the firing rate of MGB neurons in a gain control manner (Figures 4C,D), affecting the responses to all stimuli in the stimulation paradigm similarly. This “unspecific” control of the AC over the firing rate of MGB neurons did not significantly change the SSA sensitivity (quantified by a ratio of driven rates) of the majority of MGB neurons (46 of 48 neurons). Only two of the 48 neurons showed a significant reduction in SSA during AC deactivation, but these neurons still retained significant levels of SSA. Both neurons drastically increased their firing rates during AC deactivation to both stimuli, decreasing the ratio between the standard and the deviant (and therefore their SSA), as in the “iceberg effect” (Carandini and Ferster, 2000; Isaacson and Scanziani, 2011; Katzner et al., 2011. Figure 5 illustrates the “iceberg effect” mediated by GABAergic inhibition in IC neurons). These two neurons were the exception in the MGB population, where the gain changes imposed by the cortex were significant but not strong enough to cause an iceberg effect able to significantly change the level of SSA. These findings demonstrated that SSA in the MGB is neither simply inherited from the AC, nor fully generated in the first instance at the level of the AC.

Although SSA was only weakly affected by cortical deactivation, this study found an interesting relationship between SSA and the changes imposed by the AC. The gain exerted by the AC varies significantly with the level of SSA exhibited by the MGB neurons such that the facilitation exerted by the AC on MGB neurons decreases as the SSA increases (Figures 4C,D). This relationship is not dependent on the anatomical subdivision to which the MGB neurons belong, but only on their level of SSA. Hence, the AC facilitates neurons with no or low SSA, diminishing this facilitatory effect as the SSA increases. Some highly adapting neurons were even suppressed by the AC. So, although SSA in the MGB is not driven by the AC, there is an active communication between the thalamus and the cortex that is moderated according to the SSA of the individual thalamic neurons. The lack of correlation between the firing rate changes and the subdivision suggests that it is the degree of SSA rather than the localization within one or the other of the subdivisions that determines the modulatory effect of the AC on the MGB.

Although there was no significant statistical effect of subdivision on the firing rate changes, the majority of non-adapting neurons from the lemniscal MGV, a subdivision strongly driven by the corticofugal modulation originating from layer VI of A1, were mainly facilitated by the AC, as demonstrated in previous studies using AC electrical stimulation (He et al., 2002; He, 2003a,b; Yu et al., 2004). This facilitation can be achieved by direct excitation from the AC and/or by a release of inhibitory inputs from IC neurons via TRN inhibition on these inhibitory inputs. Through modulating non-adapting neurons in such a way the AC is not only facilitating the responses to the deviant stimulus but also to the standard stimulus. Therefore, we can speculate that the AC can reinforce frequently occurring signals and may contribute to a subcortical repetition enhancement effect, a phenomenon known to occur in humans to complex stimulation (Skoe and Kraus, 2010a,b). In this manner, the incoming stream is constantly being monitored, even when the stimulus is physically invariant and attention is directed elsewhere, creating predictable patterns and expectations (Winkler et al., 2009; Skoe and Kraus, 2010a,b; Skoe et al., 2013, 2014). By contrast, Antunes and Malmierca demonstrated that some high adapting neurons (i.e., CSI > 0.5) from the non-lemniscal MGB received a suppressive influence from the corticofugal pathway, presumably resulting from the strong corticofugal inhibitory effects on the non-lemniscal MGB via the TRN (Villa and Abeles, 1990; He, 2003a,b; Yu et al., 2004). Such inhibitory modulation can switch off the non-lemniscal MGB and indeed, it has been suggested as a way to functionally prepare the AC for sole processing of auditory information (Yu et al., 2004), since the non-lemniscal MGB is involved in multisensory integration (Doron et al., 2002).

Antunes and Malmierca (2011) showed that the possible inhibition driven by the corticofugal pathway did not underlie SSA in the MGB neurons, since the neurons maintained SSA during AC deactivation. Hence, if inhibition plays a role in SSA, as has been suggested in previous studies (Eytan et al., 2003; Yu et al., 2009; Richardson et al., 2011; Duque et al., 2014), other pathways should be involved, such as those coming from the IC (Peruzzi et al., 1997), the TRN-MGB connections themselves (Yu et al., 2009), as well as non-AC inputs to the TRN from basal forebrain (Hallanger and Wainer, 1988; Asanuma and Porter, 1990; Bickford et al., 1994), amygdala (Zikopoulos and Barbas, 2012), prefrontal cortex (Zikopoulos and Barbas, 2006), and GABAergic inputs from zona incerta (Cavdar et al., 2006). It may be that in the highly adaptive neurons, the suppressive effect exerted by the corticofugal pathway on the general responses of the neuron helps to increase the contrast between the standard and the deviant, as in the “iceberg effect” (Carandini and Ferster, 2000; Isaacson and Scanziani, 2011; Katzner et al., 2011. Figure 5 illustrates the “iceberg effect” mediated by GABAergic inhibition in IC neurons). This would help the AC to focus on processing the information encoded in the deviant stimulus, by reducing the responses still evoked by the commonly repeated one. It could be that the AC, together with the TRN, participates in a modulatory mechanism for a balance between excitation and inhibition that, by reducing the firing rate of the neuron, increases the contrast between the standard and the deviant, and therefore increases SSA sensitivity (Figure 5). In the same line, the fact that the highly adapting neurons receive less facilitatory influence from the cortex leads to a low discharge rate of these neurons, consequently augmenting the contrast between the standard and the deviant, thus increasing SSA. Indeed, highly adapting neurons in the MGB show lower discharge rates than non-adapting neurons (Antunes et al., 2010).

A subset of neurons had their acoustic responsiveness eliminated with cortical deactivation (four in the MGD, two in the MGV; the other was not histologically localized). These data are in agreement with the drivers and modulators hypothesis proposed by Sherman and Guillery (1998). The main corticofugal projections to the MGB arise from layer VI neurons, whose terminals are mostly small and modulatory (Rouiller and Welker, 1991, 2000; Ojima, 1994; Bajo et al., 1995; Bartlett et al., 2000; Ojima and Rouiller, 2011). In addition, a few pyramidal neurons from layer V with large terminal boutons of the driver type project to the MGD and MGV subdivisions (Rouiller and Welker, 1991, 2000; Ojima, 1994; Bajo et al., 1995; Bartlett et al., 2000; Ojima and Rouiller, 2011). The seven neurons that ceased firing during AC cooling had nonexistent or very low levels of SSA, agreeing with our main result that SSA in the MGB is not inherited from the AC.

## The effect of the corticofugal projections on collicular SSA

As previously mentioned, it has long been known that the IC receives descending projections from the AC, and therefore the responses of these IC neurons could be influenced by cortical activity (Saldaña et al., 1996; Winer et al., 1998; Malmierca and Ryugo, 2011). Although the corticocollicular projection terminates more densely on the cortical regions of the IC (Saldaña et al., 1996; Winer et al., 1998), these corticocollicular projections can also target the central nucleus directly, albeit less densely. The corticocollicular projections are excitatory (Feliciano and Potashner, 1995), however, the excitatory and inhibitory intra- and intercollicular projections (Hernandez et al., 2006) may also propagate this cortical feedback throughout the entire IC (Malmierca et al., 2005). Therefore, the corticocollicular projection may result in either an excitatory or inhibitory effect on neurons throughout the entire IC. Indeed, previous studies have demonstrated that many IC properties, even in the central nucleus of the IC, are controlled by the AC (Yan and Suga, 1998; Jen et al., 2001; Jen and Zhou, 2003; Yan et al., 2005; Nakamoto et al., 2008; Markovitz et al., 2013). Moreover, neurons showing SSA in the IC (Malmierca et al., 2009; Duque et al., 2012; Ayala and Malmierca, 2013; Ayala et al., 2013) may be under the modulation of the AC. Thus, we studied the effects of non-focal, reversible deactivation of the ipsilateral AC on the response of IC neurons that showed SSA (Anderson and Malmierca, 2013) using the same cortical cooling technique and experimental parameters as used for the MGB study (Antunes and Malmierca, 2011). As expected, the results demonstrated that the changes in the basic response properties of the IC neurons were widespread and strong in the majority of IC neurons (Figure 2). However, changes in SSA sensitivity were less common, with only about half of the neurons recorded showing a significant change in their SSA, while the other half remained unchanged (Figure 3). Thus, the effect of AC deactivation on the IC was in agreement with the MGB study (Antunes and Malmierca, 2011, 2014) for approximately half the IC neurons recorded, i.e., cortical deactivation caused a modulation in the discharge rate of MGB neurons which affected responses to both standard and deviant stimuli proportionally. However, the other 50% of IC neurons recorded showed disproportionate changes in the discharge rate to either the standard or the deviant stimulus, resulting in significant changes in SSA sensitivity which could either decrease (34% of recorded IC population) or increase (18%) (Figure 3).

This change in SSA could indicate the occurrence of a gain control modulation similar to that produced by the action of GABA_A_ mediated inhibition in the IC (Figure 5; Pérez-González et al., 2012), similar to that described in the visual cortex and compatible with the “iceberg effect” (Carandini and Ferster, 2000; Isaacson and Scanziani, 2011). Under normal conditions, the corticocollicular projections may activate intrinsic GABAergic neurons in the IC. AC deactivation will lead to subsequent increase in the activity of those neurons usually under an inhibitory influence, augmenting the ratio between the standard and deviant stimulus. The iceberg effect may potentially contribute to a significant change in SSA in either direction as a simple shift in the neuron’s spike output threshold could produce large changes in the ratio of responses to standard or deviant. However, during AC deactivation there were a few instances of dramatic changes in SSA sensitivity (for example see Figure 7 in Anderson and Malmierca (2013)), which suggests that in these cases the AC may take a more active role in response generation rather than merely adjusting the gain of IC responses.

In contrast to the MGB neurons, in the IC there was no relation between the changes imposed by the AC and the SSA exhibited by the neurons (Figures 4A,B). This is another important difference between the effect of the AC on the MGB and IC. However, this would not be totally unexpected since the corticofugal projection to the MGB and the IC arises mostly from different neuronal types located in different auditory cortical layers (Figure 1; layer V projects to IC, and layer VI to MGB; Bajo et al., 2010; Malmierca and Ryugo, 2011). Single neurons that target both the IC and MGB have not been identified (Figure 1; Wong and Kelly, 1981; Coomes and Schofield, 2004; Bajo and King, 2013).

## A synthesis on the cortical processing in the modulation of subcortical SSA

Our findings taken together demonstrate that the AC and the corticofugal pathway provides a gating or gain-control mechanism (Villa et al., 1991; He, 1997, 2003a,b; Yu et al., 2004) that indirectly modulates SSA and its dynamics in the MGB and IC neurons, rather than directly creating and transmitting this property to these auditory thalamic and midbrain nuclei. Previous studies of the auditory system support the hypothesis that SSA can be generated and driven in a bottom-up fashion, and then enhanced and modulated at the cortical level. For example, ascending sensory information is processed in parallel within the auditory system, with information from the thalamus arriving simultaneously and independently to multiple cortical areas (Imaizumi et al., 2004; Lee and Winer, 2008; Carrasco and Lomber, 2009a,b). This ascending information is then modulated by intracortical connections (Carrasco and Lomber, 2009a,b), as well as by cortico-thalamo-cortical loop projections (Bajo et al., 1995; Winer et al., 1999; Llano and Sherman, 2008). Such cortico-thalamo-cortical projections, also demonstrated in the somatosensory system (Theyel et al., 2010), are particularly interesting since they allow interconnections between the lemniscal and the non-lemniscal auditory nuclei (Bajo et al., 1995; Winer et al., 1999; Llano and Sherman, 2008). Moreover, there may even be the opportunity for intracortical interactions between the non-lemniscal and lemniscal AC fields, or indeed from the non-lemnical subcortical nuclei to the lemniscal AC. A recent study has demonstrated a bidirectional information flow from the non-lemniscal AC to the lemniscal AC (Carrasco and Lomber, 2010), therefore, the fact that A1 is the first lemniscal station in which SSA is widespread and strong (Ulanovsky et al., 2003) may result from this cortico-thalamo-cortical route, followed by a unidirectional flow of information from the secondary to the A1 (Carrasco and Lomber, 2009a,b). The thalamo-cortical projection from the medial subdivision of the MGB (MGM) to A1 is also a candidate causing an enhancement of SSA in A1 (Malmierca et al., 2002; Kimura et al., 2003; Anderson et al., 2006).

The feedback driven modulation of the subcortical nuclei that includes the MGB and IC would likely influence the transfer of ascending input to the AC (Villa et al., 1991; Luo et al., 2008), as well as the corticocortical processing itself, as demonstrated in other sensory systems (Sillito et al., 2006; Theyel et al., 2010). Recent studies suggest that the corticofugal system can participate in a gain control process that leads to improved coding of salient stimuli, and possibly underlies auditory attention (He, 2003a,b) and learning-induced plasticity mechanisms (Bajo et al., 2010; Skoe et al., 2013). Conversely, the increased coupling of cortical and thalamic activity may amplify the effectiveness of a particular feature of the external sensory input, allowing its detection and binding to higher cognitive processing (Villa et al., 1999). Our results are consistent with the role of the corticofugal pathway in scaling the sensitivity of MGB and IC neurons to its driving inputs by controlling their gain. SSA would therefore be generated in a bottom-up fashion, as a pre-attentive gating involved in reducing sensory input to behaviorally relevant aspects.

The differential influence of the descending cortical projection on SSA in IC and MGB may reflect a method of statistical refinement in the process of bottom-up transmission based on the suggestion that there is a redundancy reduction throughout successive levels (Schwartz and Simoncelli, 2001; Chechik et al., 2006). For example, previous measurements of information content and stimulus-induced redundancy from neurons in the IC, MGB and primary AC in response to natural stimuli, suggest stimulus identity was reduced in the AC and MGB neurons compared to those in IC (Chechik et al., 2006). Thus, redundancy reduction may be a generic organizing principle of neural systems, and a potential role for the descending cortical projection would be to make gross changes at the lowest possible level of the pathway. Therefore, if SSA to frequency deviants is generated in the IC (and/or below) the greatest AC modulation will be at this lowest level, and then as SSA is propagated up the auditory pathway (MGB) it would only require minor modifications. Since the rat IC contains about 350,000 neurons while MGB only 65,000 (i.e., less than 20% of the total of IC neurons; Kulesza et al., 2002) individual neurons in the MGB will be integrating over more inputs than individual IC neurons, and hence, the role of the MGB neurons may be to combine the adaptive properties received across a range of inputs, rather than generate SSA de-novo which would subsequently require greater cortical intervention.

## Stimulus-specific adaptation, mismatch negativity and predictive coding: the interaction between bottom-up and top-down processing

The mismatch negativity (MMN) is an evoked scalp potential elicited by rare events embedded in a series of frequently repeating events (Näätänen et al., 1978). Since SSA is a decrease, or even a total cessation, in the response of a single neuron to a repeated stimulus, which recovers on the presentation of another, new stimulus (Ulanovsky et al., 2003), it resembles in many aspects the phenomenon of MMN, and it has been proposed to be a precursor to the generation of deviance detection (Nelken and Ulanovsky, 2007; Malmierca et al., 2014).

**Figure 6:**
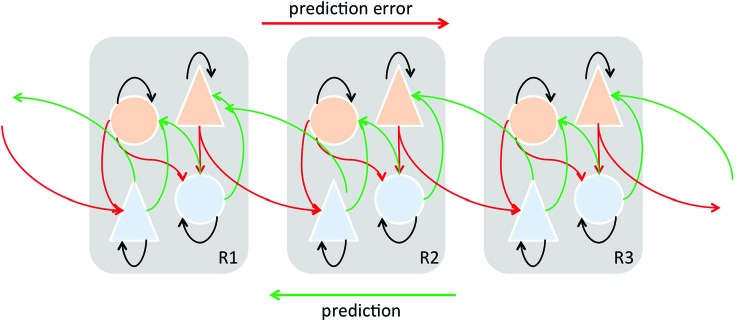
**Simplified schematic diagram detailing the neuronal architectures that might encode a density on the states of a hierarchical dynamic model of predictive coding by Friston (2005, 2009)**. State units are in light blue and error-units in orange. This shows the speculative cells of origin of forward driving connections that convey prediction error from a lower area to a higher area and the backward connections that construct predictions. These predictions try to explain prediction error in lower levels. In this scheme, the sources of forward and backward connections are superficial and deep pyramidal cells, respectively. In the original model by Friston (2005, 2009) subcortical regions may contribute to the model as well. “Predictions” and “prediction errors” are sent and received from each level in the hierarchy. Feed-forward signals conveying prediction errors originate in superficial layers and terminate in deep (infragranular) layers of their targets, are mediated by GABA and fast AMPA receptor kinetics and associated with gamma-band oscillations. On the other hand, feedback signals conveying predictions originate in deep layers and project to superficial layers, are mediated by slow NMDA receptor kinetics and associated with beta-band oscillations Adapted and modified from Friston (2005, 2009), Seth et al. (2012).

In MMN paradigms, short term predictive representations of environmental regularities are thought to be formed based on the observed likelihood of frequently repeating events (standard). Implicitly learned statistical regularities serve as a basis to automatically detect rare events (deviant) which do not match predictions. Recent modeling studies (Lieder et al., 2013) suggested that the MMN reflects approximate Bayesian learning of sensory regularities, and that the MMN-generating process adjusts a probabilistic model of the environment according to mismatch responses (prediction errors). Thus, the *MMN response is now widely considered as a perceptual prediction error signal* (Friston, 2005; Garrido et al., 2008, 2009; den Ouden et al., 2012), which can be considered a member of a family of prediction errors, which include perceptual, higher cognitive, and motivational prediction errors (Figure 6).

The MMN response is widely considered as a perceptual prediction error signal (Friston, 2005; Winkler, 2007; Garrido et al., 2008, 2009; den Ouden et al., 2012; Winkler et al., 2012; Fishman, 2014) that results from the comparison between the actual sensory input and a memory trace so-called prediction encoded in top-down activity (Rao and Ballard, 1999; Yuille and Kersten, 2006). A prediction error would arise when there is a mismatch between the predicted and the actual sensory input (Figure 6). According to the hierarchical predictive coding framework, veridical prediction is supported by neural processes optimizing probabilistic representations of the causes of sensory inputs (Figure 6; Friston, 2010). SSA as an adaptation process may in its own way improve the neural coding efficiency by reducing the response to potentially redundant information (in a similar manner to redundancy reduction as suggested by Chechik et al., 2006). Moreover, as we have reviewed previously, there are plenty of opportunities for a continuous interaction between the top-down flow of predictions and bottom-up flow of prediction errors to enable a continual updating and optimizing of the internal sensory representation. We do not currently know the detailed organization of the inputs received by those subcortical neurons showing significant SSA, but the fact that auditory cortical inputs project more densely to those areas populated by neurons showing SSA, especially in the IC (but also in the MGB), suggests neurons showing SSA may be key players in the integration of descending information with the incoming information from lower areas. Thus, when a prediction error occurs the lower areas would return the ensuing prediction error by means of forward connections and the corticofugal projections would drive the neuronal activity along different neuronal stages to adjust and update the sensory representation (Winkler et al., 2009). The top-down adjustment would be through direct or indirect inputs which in turn would project back to higher auditory centers (Figure 6). Also, it is important to highlight that the sensory representation requires fast *on-line* adjustments on the neuronal activity. In agreement with this, the corticofugal modulation on IC neurons has been demonstrated to exert short-term plastic reorganization in the frequency domain (i.e, Suga and Ma, 2003) and on SSA responses (Anderson and Malmierca, 2013). For example, as previously demonstrated, the cortical cells projecting to the IC arise mainly from pyramidal neurons in layer V (Games and Winer, 1988; Winer and Prieto, 2001; Doucet et al., 2003; Malmierca and Ryugo, 2011) and use glutamate as neurotransmitter (Feliciano and Potashner, 1995). Since predictive coding is based on NMDA-dependent synaptic plasticity and its regulation by other neuromodulators (Friston, 2005), future pharmacological studies will undoubtedly help to disentangle which role synaptic plasticity plays that may underlie the neurobiological mechanisms of MMN as well as the SSA responses.

## Concluding remarks

In summary, here we have reviewed the neuronal and anatomical basis that may underlie SSA, MMN and predictive coding. The corticofugal projections arising from the AC may be much more important than previously estimated since they may play a key role linking these fields to the concept of mental models (Craik, 1943; Bendixen et al., 2012), allowing us to foresee the consequences of a given action and enabling us to prepare for future events, such as the rain that is to be expected, or the bad mood of our partner or boss. Thus, consciously or unconsciously we can then take appropriate action to prevent undesirable and unpleasant consequences, e.g., getting wet by the rain, or even change the future, e.g., to take proactive action to ensure the bad mood of our partner or supervisor will not occur. These examples illustrate that behavior is optimized when we can predict the dimensions of a stimulus (Kotz et al., 2014). This is the basis of the huge flexibility underlying our interactions with our physical and social environment.

## Conflict of interest statement

The authors declare that the research was conducted in the absence of any commercial or financial relationships that could be construed as a potential conflict of interest.
